# Supraoptimal Iron Nutrition of *Brassica napus* Plants Suppresses the Iron Uptake of Chloroplasts by Down-Regulating Chloroplast Ferric Chelate Reductase

**DOI:** 10.3389/fpls.2021.658987

**Published:** 2021-05-20

**Authors:** Máté Sági-Kazár, Helga Zelenyánszki, Brigitta Müller, Barnabás Cseh, Balázs Gyuris, Sophie Z. Farkas, Ferenc Fodor, Brigitta Tóth, Béla Kovács, Anna Koncz, Tamás Visnovitz, Edit I. Buzás, Barbara Bánkúti, Ferenc Bánáti, Kálmán Szenthe, Ádám Solti

**Affiliations:** ^1^Department of Plant Physiology and Molecular Plant Biology, Institute of Biology, ELTE Eötvös Loránd University, Budapest, Hungary; ^2^Institute of Food Science, Faculty of Agricultural and Food Sciences and Environmental Management, University of Debrecen, Debrecen, Hungary; ^3^Department of Genetics, Cell- and Immunobiology, Semmelweis University, Budapest, Hungary; ^4^MTA-SE Immune-Proteogenomics Extracellular Vesicle Research Group, Budapest, Hungary; ^5^HCEMM-SE Extracellular Vesicle Research Group, Budapest, Hungary; ^6^RT-Europe Non-profit Research Ltd., Mosonmagyaróvár, Hungary; ^7^Carlsbad Research Organization Center Ltd., Újrónafõ, Hungary

**Keywords:** *Brassica napus*, chloroplast, FCR activity, FRO7, iron allocation, iron nutrition, iron uptake

## Abstract

Iron (Fe) is an essential micronutrient for plants. Due to the requirement for Fe of the photosynthetic apparatus, the majority of shoot Fe content is localised in the chloroplasts of mesophyll cells. The reduction-based mechanism has prime importance in the Fe uptake of chloroplasts operated by Ferric Reductase Oxidase 7 (FRO7) in the inner chloroplast envelope membrane. Orthologue of *Arabidopsis thaliana FRO7* was identified in the *Brassica napus* genome. GFP-tagged construct of BnFRO7 showed integration to the chloroplast. The time-scale expression pattern of *BnFRO7* was studied under three different conditions: deficient, optimal, and supraoptimal Fe nutrition in both leaves developed before and during the treatments. Although Fe deficiency has not increased *BnFRO7* expression, the slight overload in the Fe nutrition of the plants induced significant alterations in both the pattern and extent of its expression leading to the transcript level suppression. The Fe uptake of isolated chloroplasts decreased under both Fe deficiency and supraoptimal Fe nutrition. Since the enzymatic characteristics of the ferric chelate reductase (FCR) activity of purified chloroplast inner envelope membranes showed a significant loss for the substrate affinity with an unchanged saturation rate, protein level regulation mechanisms are suggested to be also involved in the suppression of the reduction-based Fe uptake of chloroplasts together with the saturation of the requirement for Fe.

## Introduction

Iron (Fe)-dependent redox reactions occur in a wide range of plant metabolic processes (Connorton et al., 2017). In plants, 80–90% of cellular Fe is found in chloroplasts (Hantzis et al., 2018), localised mainly in photosystem I and under the excess of Fe or if the photosynthetic apparatus decomposes, also in ferritin (Chen et al., 2019). For the operation of a single photosynthetic electron transport chain, a total of 22 Fe atoms are needed in heme groups of photosystem I and cytochrome *b_6_/f*, in Fe–S clusters of PSI, cytochrome *b_6_/f* and ferredoxin and as non-heme ferro-cofactors in photosystem II [for review, see Schmidt et al. (2020)]. Thus, incorporation of Fe into Fe–S clusters has a priority that is coupled to the Fe acquisition of chloroplasts (Solti et al., 2012, 2016). Fe is also a key cofactor in chlorophyll biosynthesis as Fe^2+^ cofactor of Mg-protoporphyrin IX monomethyl ester cyclase (Spiller et al., 1982) and Fe–S cofactor of chlorophyll *a* oxidase (Hu et al., 2017). Limited availability of Fe for chloroplasts leads to reduced photosynthetic activity. In consequence, Fe deficiency causes chlorosis on the leaves and a significant decrease in biomass production (Hantzis et al., 2018).

In contrast to the well-characterised root Fe homeostasis (Rodríguez-Celma et al., 2019; Schwarz and Bauer, 2020), the regulation of Fe metabolism in mesophyll cells and in their organelles has not been fully revealed yet (Vigani et al., 2019; Schwarz and Bauer, 2020). Chloroplasts operate a reduction-based Fe uptake strategy (Solti et al., 2014; Müller et al., 2019). This machinery proposed to include the chloroplast inner envelope (cIE) membrane proteins PIC1/TIC21 and FRO7. The role of other Fe uptake and homeostasis-related components NiCo, MAR1/IREG3, YSL4/6, and MFL1 is much less characterised (for review, see Vigani et al., 2019). Recently, ABCI11/NAP14 was shown to play a crucial role in Fe homeostasis (Voith von Voithenberg et al., 2019). Furthermore, recently, NiCo was indicated not participating in the uptake together with PIC1 in an exclusive manner (Pham et al., 2020). Chloroplast NEET protein exports Fe–S clusters to cytoplasmic Fe–S proteins. Disrupting NEET function results in enhanced reactive oxygen species (ROS) and Fe over-accumulation in chloroplasts (Zandalinas et al., 2019). RNAi of At-NEET resulted in developmental retardation, increased senescence and elevated sensitivity to low and decreased sensitivity to high Fe nutrition (Nechushtai et al., 2012; Lu, 2018). Indeed, little is known on the Fe release from chloroplasts to date (Vigani et al., 2019; Pham et al., 2020). Although Fe is an essential micronutrient for all living things, free ferrous Fe ions can catalyse Fenton reactions (Halliwell and Gutteridge, 1992); thus, accumulation of Fe in supraoptimal concentration can lead to the production of ROS (Ravet et al., 2009; Briat et al., 2010). Therefore, Fe acquisition, translocation and storage are strictly regulated in plants (Nikolić and Pavlović, 2018). In a previous study, internal Fe concentration of the chloroplasts was found suppressing the Fe uptake of chloroplasts over a certain concentration limit (Solti et al., 2012). Nevertheless, the background of this decreased uptake was not revealed.

Ferric Reductase Oxidase (FRO) family proteins are key components of the reduction-based Fe transport strategy in plants, fungi (FRE family proteins of yeast), and mammals (Jeong and Connolly, 2009). In *Arabidopsis*, eight FRO family proteins are encoded in the genome that are targeted to the root and shoot plasma membrane, to mitochondrial envelope and to the inner envelope of chloroplasts (Jain et al., 2014; Solti et al., 2014). Except on a recent report on *Oryza sativa* OsFRO1 (Li et al., 2019), no FRO family proteins were found being targeted to the vacuolar membrane. Moreover, there is a variance among species in the number of FRO family members responsible for the ferric chelate reductase (FCR) activity of certain membranes. Although in *Arabidopsis* a single protein FRO2 is thought to perform root FCR activity (Robinson et al., 1999), there are three root plasma membrane-directed FRO family proteins in *Cucumis sativus* (Marastoni et al., 2019). Satbhai et al. (2017) also reported a significant variation of the non-coding region of *FRO2* locus in *Arabidopsis* across accessions resulting in a variation in responses to Fe limitation. A deep understanding of the regulation of Fe homeostasis at plant and at intracellular levels has primary role to drive biofortification efforts.

Iron uptake of chloroplasts operates by the reduction-based strategy (Vigani et al., 2019; Kroh and Pilon, 2020b), where the reducing power originates from NADPH produced by the photosynthetic apparatus (Bughio et al., 1997; Solti et al., 2014). In *Arabidopsis*, FRO7 is responsible for the FCR activity of chloroplasts (Jeong et al., 2008). In *fro7* mutants, photosynthetic activity and the Fe content of the chloroplasts significantly decreased in comparison to the wild type, but FRO7 itself was not characterised so far. Moreover, alterations in the expression and activity of FRO7 among developmental stages have not been resolved yet. Since Fe uptake of chloroplasts and thus FRO7 is essential in the development of the photosynthetic apparatus, but Fe uptake itself depends on the photosynthetic function, the connection between chloroplast Fe acquisition and Fe deficiency responses is reciprocal. Thus, here we aimed to reveal the dependence of FRO7 on the Fe nutrition and developmental status using a *Brassica* model, close relative to *Arabidopsis*.

## Materials and Methods

### Plant Material

Oilseed rape (*Brassica napus* L. cv. DK Exquisite) seeds were germinated on moderate light (less than 50 μmol photons m^–2^ s^–1^ photosynthetic photon flux density) and planted directly into nutrient solution [half-strength Hoagland solution: 2.5 mM Ca(NO_3_)_2_, 2.5 mM KNO_3_, 1.0 mM MgSO_4_, 0.5 mM KH_2_PO_4_, 0.16 μM CuSO_4_, 9.2 μM MnCl_2_, 0.38 μM ZnSO_4_, 0.24 μM Na_2_MoO_4_, 23.12 μM H_3_BO_3_, and 20 μM Fe(III)-EDTA as optimal Fe source] in 12 L plastic buckets for 10 seedlings in one bucket. Plants were grown in a climate controlled chamber (14 h 120 μmol photons m^–2^ s^–1^ photosynthetic photon flux density illumination/10 h dark periods) at 70/85% relative humidity and 22/24°C. Precultivation lasted until seedlings reached a four-leaf stage, then nutrient solutions were changed and treatments were applied.

To induce Fe deficiency (dFe), a group of precultivated four-leaf-stage seedlings were transferred to Fe-free nutrient solution supplied with 0.5% (m/V) CaCO_3_. Supraoptimal (sFe) Fe supply was achieved applying 100 μM Fe(III)-EDTA (five times higher concentration than optimal Fe nutrition). Plants of optimal Fe nutrition were further grown on 20 μM Fe(III)-EDTA (oFe). Leaves that emerged after the start of treatment (5th leaves ongoing) were distinguished. For chloroplast envelope membrane isolation and chloroplast Fe uptake studies, leaves that emerged after the start of treatment were used. For gene expression analyses, 4th (emerged before the start of treatment) and 6th (emerged after the start of treatment) were collected.

### Chloroplast Isolation and Determination of Chloroplast Intactness

Leaves were harvested and homogenised with a Waring Blender for 5 s in isolation buffer containing 50 mM HEPES–KOH, pH 7.0, 330 mM sorbitol, 2 mM EDTA, 2 mM MgCl_2_, 0.1% (w/V) BSA, and 0.1% (w/V) Na ascorbate. The homogenate was filtered through four layers of gauze and two layers of Miracloth^TM^ (Calbiochem–Novabiochem, San Diego, CA, United States) and centrifuged at 1,600 × *g* for 5 min. All centrifugation steps were carried out in a swing-out rotor at 4°C. The pellet was resuspended in a washing buffer containing 50 mM HEPES–KOH, pH 7.0, 330 mM sorbitol, 2 mM EDTA and 2 mM MgCl_2_, then layered on a three-step sucrose gradient [50 mM HEPES–KOH, pH 7.0, 20/45/60% (m/V) sucrose, 2 mM EDTA, 2 mM MgCl_2_] and centrifuged at 2,500 × *g* for 20 min. Intact chloroplasts were collected from the 45/60% sucrose phase transition. Following a 5–10-fold dilution by adding washing buffer, the plastid fraction was centrifuged at 2,500 × *g* for 5 min, and the pellet was resuspended in washing buffer. Chloroplast density was determined by counting in a Bürker chamber using a Nikon Optiphot-2 microscope. The intactness of chloroplasts was determined based on the RbcL/apoLHCII ratio in the chloroplast samples, according to Müller et al. (2019).

### Chloroplast Fe Uptake Assays

Intact chloroplast suspensions were diluted by adding washing buffer to 100 μg chlorophyll (Chl) ml^–1^. The Chl content of chloroplasts was determined in 80% (V/V) acetone extracts using a UV–VIS spectrophotometer (Shimadzu, Kyoto, Japan) using the absorption coefficients of Porra et al. (1989). Fe uptake assay medium contained 0.5 ml of chloroplast suspension with altering concentrations of Fe(III)-citrate as Fe source. Fe uptake was initiated by the illumination of the solution with 120 μmol photons m^–2^ s^–1^ actinic light (Philips HPI-T Plus, 250 W metal halogen lamp) and terminated by placing the samples on ice and in darkness after 30 min incubation. As for terminating the assay, chloroplasts were immediately centrifuged at 2,500 × *g* for 5 min at 4°C in a swing-out rotor. Pelleted chloroplasts were washed with 0.25 ml washing buffer containing 2 mM (m/V) EDTA and pelleted again. Pellets were resuspended in washing buffer containing 1% (m/V) SDS and 1% (m/V) DTT and solubilised at room temperature for 30 min. Starch was removed from samples by centrifugation at 10,000 × *g* for 5 min. Fe was reduced and complexed in the samples to ferrous Fe by adding 300 μM ascorbic acid and 300 μM bathophenanthroline disulfonate disodium salt (BPDS; Sigma), respectively. Reduced Fe content was measured by UV–VIS 2600 spectrophotometer using Super Micro Black Cells (Shimadzu, Japan) at 535 nm as [Fe-(tri)bathophenanthroline disulfonate]^4–^ and an absorption coefficient of 22.14 mM^–1^ cm^–1^ (Smith et al., 1952).

### Isolation of Chloroplast Envelope Membranes

Chloroplast envelope membranes were purified according to Solti et al. (2014) with slight modifications adopting the method on a *Brassica* model. Washed intact chloroplasts were subjected to envelope membrane vesicle isolation. Chloroplasts were suspended in TE buffer (10 mM Tricin–KOH, pH 7.8, 2 mM EDTA) with 0.6 M sucrose and then broken in three freeze/thaw (−20/0°C) cycles. Following the last thaw phase, the suspension was diluted three times with TE buffer to achieve 0.2 M sucrose content and incubated for 1 h on ice. Thylakoid membranes were removed from the suspension by centrifugation at 4,500 × *g* for 15 min in a swing-out rotor at 4°C, whereas envelope membranes remained in the supernatant. The supernatant was collected and centrifuged at 25,000 × *g* for 60 min using a swing-out rotor (Sw40Ti) in a Beckman L7 ultracentrifuge (Beckman Coulter Inc., Brea, CA, United States). The pellet was resuspended in TE buffer containing 0.2 M sucrose and transferred to a sucrose gradient. The gradient was prepared from TE buffer with 1/0.8/0.45 M sucrose with equal volumes. Through ultra-centrifuging at 140,000 × *g* for 120 min in a swing-out rotor, envelope and thylakoid membranes were separated from each other. Envelope membrane fractions were collected from the 1/0.8 M and the 0.8/0.45 M gradient interface. Inner envelope membrane vesicles are found in the former, whereas the latter contains a mix of inner and outer envelope membrane vesicles. The membrane fractions were diluted with TE buffer and ultra-centrifuged again at 40,000 × *g* for 75 min in a swing-out rotor. Pellets were resuspended in TE buffer and stored at −80°C until use.

To control the purity of the isolated membrane fractions, samples were solubilised in 62.5 mM Tris–HCl, pH 6.8, 2% (m/V) SDS, 2% (m/V) dithiothreitol, 10% (V/V) glycerol and 0.001% (m/V) bromophenol blue at room temperature for 30 min. Proteins were separated according to Laemmli (1970) but in 10–18% gradient polyacrylamide gels in a MiniProtean apparatus (Bio-Rad Laboratories Inc., Hercules, CA, United States) using a constant current of 20 mA per gel at 6°C. The protein concentration of samples was determined by comparing the area density with that of a standard mixture using Phoretix 4.01 software (Phoretix International, Newcastle upon Tyne, United Kingdom). In order to check the purity of the isolated membrane fractions, protein blots were carried out against a chloroplast triose-phosphate translocator (cTPT, a cIE marker; Lundmark et al., 2006) and inner envelope protein 37 (IEP37, a cIE marker) and light-harvesting complex apoproteins (apoLHC, a thylakoid marker). Membrane proteins separated by SDS-PAGE were transferred to Amersham^TM^ Protran^TM^ premium 0.2 μm NC (GE Healthcare, IL, United States) nitrocellulose membranes in 25 mM Tris, pH 8.3, 192 mM glycine, 20% (V/V) methanol, and 0.02% (m/V) SDS at 4°C using 90 V constant voltage (<0.4 A) for 3 h. Membranes were decorated with rabbit polyclonal antibodies against apoLHCII (a kind gift from Dr. Udo Johanningmeier, Bochum University, Germany), IEP37 (a kind gift from Prof. Katrin Philippar, Saarbrücken University, Germany) and cTPT (Agrisera AG, Vännäs, Sweden). Antibodies were dissolved in 20 mM Tris–HCl, pH 7.5, 0.15 M NaCl and 1% (m/V) gelatine following the manufacturer’s instructions. Horseradish peroxidase (HRP)-conjugated goat anti-rabbit IgG (Bio-Rad) was used according to the manufacturer’s instructions.

### FCR Activity of cIE Membrane Vesicles

Ferric chelate reductase (EC 1.16.1.9) activity was measured according to Solti et al. (2014). Chloroplast envelope vesicles equivalent to 10–20 μg protein suspended in TE buffer were mixed with 10 mM NADPH and 10 mM FAD (Sigma). Vesicles were loaded with NADPH and FAD by a freeze–thaw cycle (−20/0°C), to ensure only right-side-out chloroplast envelope membrane vesicles could participate in the FCR reactions. The final reaction mixture consisted of 330 mM sorbitol, 2 mM MgCl_2_, 100 μM FAD, 100 μM NADPH, and 300 μM BPDS and envelope vesicles equivalent to 10–12 μg total proteins. Fe(III)-EDTA was used as substrate in altering concentrations from 10 to 100 μM external Fe concentration. The reaction was initiated by the addition of Fe(III)-EDTA to the mixture. To eliminate the background reaction of NADPH with Fe(III)-EDTA, a blank reaction mix was used without envelope vesicles. Although FAD and NADPH were present both in the vesicles and in the reaction mixture, Fe(III)-EDTA and BPDS were absent in the vesicles; thus, the FCR reaction could only be mediated by right-side-out vesicles. FCR assay was monitored by UV–VIS 2600 spectrophotometer using Super Micro Black Cells (Shimadzu, Japan) at 535 nm for 40 min. The reaction rate was calculated from the linear phase of the increase in the absorption at 535 nm using an absorption coefficient of 22.14 mM^–1^ cm^–1^ (Smith et al., 1952).

### Determining the Concentration of Elements

Leaf samples collected parallel with samples for gene expression analysis were dried for a week at 60°C. Samples were powdered then digested by HNO_3_ for 30 min at 60°C and bleached by H_2_O_2_ for 90 min at 120°C. After filtration through MN 640 W paper, element contents were measured by inductively coupled plasma mass spectrometry (ICP-MS; Thermo Fisher, United States).

### Identification of *Brassica* Orthologue of the *Arabidopsis FRO7* Gene

To identify putative homologs of *AtFRO7* (*At5g49740*) in *B. napus*, protein sequence blasting was performed in the Brassica database^1^. A reciprocal blast of the identified sequence was performed against *Arabidopsis* transcripts in the TAIR database^2^. A gene encoding *Brassica* orthologue of the *Arabidopsis* query was identified in the *B. napus* genome originated from the *Brassica rapa* parental genome as *FRO7* orthologue [*GSBRNA2T00048061001* (BnaAnng20940D); score: 1,635, e^0^; 3.976 kbp sequence on chrAnn_random from 23,224,275 to 23,228,251 (plus strand); reciprocal best hit: *At5g49740.1*; score: 2,561, e^0^]. Protein sequences of AtFRO7 and BnFFO7 were aligned in the online CLUSTAL Omega program^3^. Sequence identity and similarity were calculated using the Ident and Sim online tool^4^. To identify a chloroplast transit peptide, sequences were further analysed in the ChloroP 1.1 Server^5^.

### Transient Expression and Localisation of FRO7-GFP

To validate the identification of *Brassica FRO7*, a GFP-labelled fusion protein was expressed in leaf tissue. The *FRO7-GFP* transgene and the native promoter region of *Brassica FRO7* (1,875 bp upstream to the start codon of *GSBRNA2T00048061001*) were cloned into the pCambia2301 vector. Application of primers used for cloning of the promoter sequence [fw: 5′-GGTACCCGTTACTCTCTCTGTGTAGC-3′ (5′ *Kpn*I restriction site) and rev: 5′-GAAAAAGTATGTAGATCTTATGTCGG-3′ (covering *Bgl*II restriction site)] resulted in a 1,900 bp product. Transient expression studies were carried out by infiltration of AGL1 strain of *Rhizobium radiobacter* (formerly *Agrobacterium tumefaciens*) carrying the gene of interest into young but already developed leaves of common bean (*Phaseolus vulgaris* L. var. *nanus* cv. Borlotto). Agroinfiltration was performed as described by Goodin et al. (2002). Transient presence of GFP fluorescence was detected by illuminating infiltrated leaves by IORodeo Midi blue LED transilluminator [Shenzhen OVTAI Optoelectronics Co., Ltd., China; *E* = 474 (±12) nm]. GFP fluorescence was detected as *F* = 510 nm emitting dots visually. Intact chloroplasts were isolated and purified from positive spots of bean leaves 3–5 days after infiltration according to the method applied for *B. napus* chloroplasts. Isolated chloroplasts were fixed in 4% (m/V) paraformaldehyde dissolved in phosphate-buffered saline (PBS; 137 mM NaCl, 2.7 mM KCl, 10 mM Na_2_HPO_4_, 1.8 mM KH_2_PO_4_, pH 7.4, supplemented with NaCl to a final concentration of 300 mM to ensure isosmotic environment) for 20 min. Fixed chloroplasts were blocked and permeabilised with 5% (m/V) BSA and 0.5% (V/V) Tween-20 in PBS and then incubated with GFP specific antibody (Sigma-Aldrich; SAB4301138) diluted 1:200 for 16 h. Anti-rabbit-IgG Atto 550 antibody (Sigma-Aldrich; 43328) was used in 1:500 dilution in PBS with 1% (m/V) BSA for 1 h at 37°C. Chloroplast suspension was air-dried to the slides (2 min at 65°C), and then coverslips were mounted with Prolong Diamond (Invitrogen). Samples were examined with a Leica SP8 laser-confocal microscope. Atto 550 dye and Chls were excited at 552 and 638 nm and detected at 645–790 and 570–600 nm ranges, respectively. For image analysis, LAS X (Leica) software was used.

### Total RNA Extraction and cDNA Synthesis

To isolate total RNAs, approximately 80 mg of leaf tissue was homogenised in 1 ml of TRI Reagent^®^ (Sigma). Total RNA fraction was separated with 0.2 ml chloroform. Following a 15 s shaking and 15 min incubation at room temperature, the mixture was centrifuged at 12,000 × *g* for 15 min at room temperature. The supernatant was incubated at −20°C for 20 min with 0.5 ml isopropanol. Nucleic acids were pelleted out by centrifugation at 18,000 × *g* for 20 min at 4°C. The pellet was washed by 70% ethanol twice and dried at room temperature. Nucleic acids were resolved in 50 μl diethyl pyrocarbonate-treated water. Residual genomic DNA contamination was digested by RNase free DNase I (Thermo Fisher Scientific). RNA concentration and purity were checked by Nanodrop ND-1000 spectrophotometer (Thermo Fisher Scientific). Reverse transcription of the total RNA pool by RevertAid Reverse Transcriptase (Thermo Fisher Scientific) was performed at 42°C for 45 min using random hexamer oligonucleotides. The reaction was stopped by incubation at 70°C for 10 min. Until further application, cDNA libraries were stored at −80°C.

### Relative Transcript Analysis

To represent accurately and analyse reliably the expression of the targets, a robust normalisation of quantitative reverse transcription-polymerase chain reaction (qRT-PCR) data with suitable internal control genes was performed. As reference genes for qPCR studies, *18S rRNA* (KT225373), β-*tubulin* (XM_009125342.1) and *EF1*α (XM_009149797.1) coding sequences were used based on the NCBI database and tested for expression as in Pham et al. (2020). The transcript sequence of SUFB component ABCI8 of *B. napus* was accessed as XM_013855705.2 in NCBI. Primers are listed in Table 1. The specificity of each primer was verified by a sharp peak during melt curve analyses and agarose gel electrophoresis of PCR products. Single PCR products of the expected size were amplified with all target primer sequences with optimal annealing temperature and primer concentration. The efficiency of primers was estimated based on standard curves using seven points of twofold serial dilutions of cDNA template. Based on the slope of a linear regression model, the efficiency of each gene ranged from 1.87 to 1.96.

**TABLE 1:** Oligonucleotide primers used in the expression analysis.

Gene (GenBank accession, *Arabidopsis* orthologue)	Primer sequences (5′–3′)		T_*m*_ (°C)	Product (bp)
*BnFRO7* (*BnaAnng20940D*, *At5g49740*)	fw	GGTGTTCGCTAAGAAGAAGATATCG	57.5	172
	rev	GTCAAGATCCCTCATGGTATATGC		
*BnABCI8* (*XM_013855705.2*, *At4g04770*)	fw	GGGTATCTCGGCTGGCAACT	57	163
	rev	GGCTGATGGGTTCTTAACCTGGAT		
*18s RNA* (*KT225373*, *At2g16590*)	fw	GCATTCGTATTTCATAGTCAGAGGTG	61	192
	rev	CGGAGTCCTAAAAGCAACATCC		
β*-tubulin* (*XM_009125342.1*, *At4g20890*)	fw	TCSATCCAGGARATGTTCAGG	59	148
	rev	ACTCTGCAACAAGATCATTCATG		
*EF1*α (*XM_009149797.1*, *At1g07940*)	fw	CAGATCAACGAGCCAAAGAGG	56	120
	rev	CTTGAGCATACCGGTCTCAAC		

*Forward (fw) and reverse (rev) primers were applied on the annealing temperatures (T_m_) determined in preliminary studies. Amplification resulted in the accumulation of products (size is given in base pairs; bp).*

Relative expression analyses were performed by StepOnePlus Real-Time PCR system (Thermo Fisher Scientific) with StepOne^TM^ v.2.2.3 software. Amplification intensity was followed by SYBR Green (Luminaris Colour HiGreen High ROX; Thermo Fisher Scientific). The qRT-PCR program set up was: 50°C for 2 min (predigesting), 95°C for 10 min (initial denaturation), 40 cycles at 95°C for 15 s (denaturation), T_*m*_ for 30 s (annealing) and 72°C for 30 s (extension) and terminated by a final melt curve analysis. All cDNA samples were freshly diluted before qPCR reactions. Three technical and four biological parallels were analysed, and relative normalised gene expression was calculated according to Pfaffl (2001).

### Statistical Analysis

Chloroplast Fe uptake was performed in triplicates in 5–6 independent biological repetitions. FCR assays were performed in 3 × 3 (technical × biological) repetitions. Two parallel RNA samples (technical replicates) were isolated from three independent experiments (biological replicates). qRT-PCR analysis of genes and samples was processed in technical triplicates to confirm the stable expression of the gene of interest. To compare multiple treatments, one-way ANOVAs with Tukey–Kramer *post hoc* tests were performed on data using InStat v. 3.00 (GraphPad Software, Inc., San Diego, CA, United States). The term “significantly different” means that the similarity of samples is *P* < 0.05. Origin v. 6.01 (Origin Lab Co., Northampton, MA, United States) was used to fit mathematical functions on data points. Boltzmann’s function was used for K_*M*_ and v_*max*_ calculations.

## Results

### Fe Content of Leaves

**FIGURE 1:**
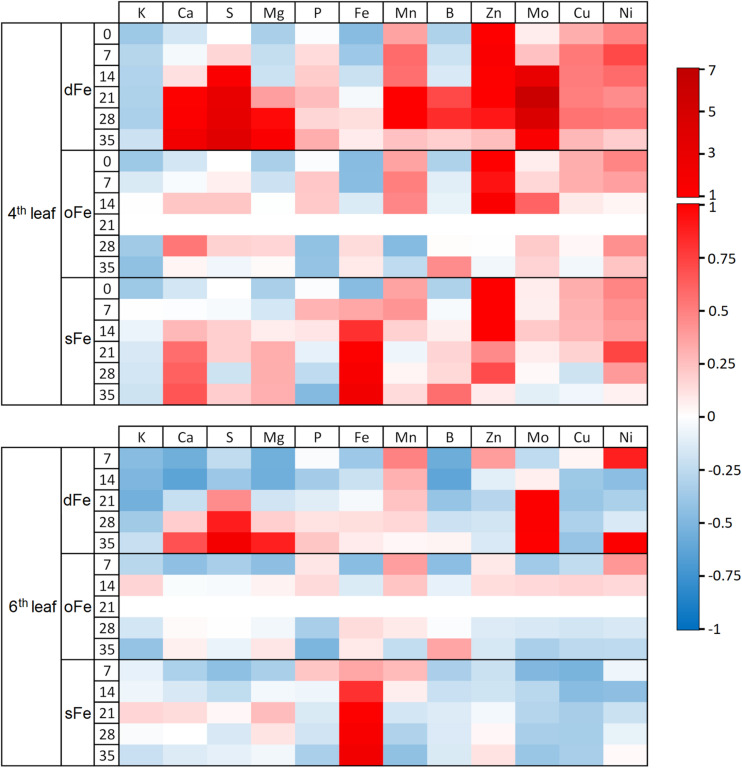
Heat map of the element accumulation of the 4th and 6th leaves of plants grown on deficient (dFe), optimal (oFe) and supraoptimal (sFe) Fe supply. Heat map plot indicates the concentration of elements as deviance from the total corresponding accumulation in oFe leaves at the 21st day of treatment (zero deviance).

Altered Fe nutrition led to differences in the accumulation of mineral nutrients in leaves (Figure 1). Regarding the 4th leaves, dFe treatment led to an accumulation of among others S, Mn, and Mo. Moreover, the pattern of accumulation changed during the time of treatment: Cs, Mg, and B accumulation was lower in the initial, but higher in the terminal phase of treatment than the corresponding oFe plants. In sFe plants, inhibition of the accumulation of *P* was among the most significant alterations compared to that in oFe plants. Regarding the 6th leaves, the effect of dFe treatment was the most pronounced in the accumulation of S and Mo, and slight accumulation was found for Ca and Mg at the very end of the time of treatment. In turn, especially during the leaf development, accumulation of K, Ca, Mg, and B was retarded compared to the corresponding oFe leaves. In sFe plants, in parallel to the accumulation of Fe, the Mo and Cu contents of leaves were retarded significantly compared to the corresponding oFe leaves.

**FIGURE 2:**
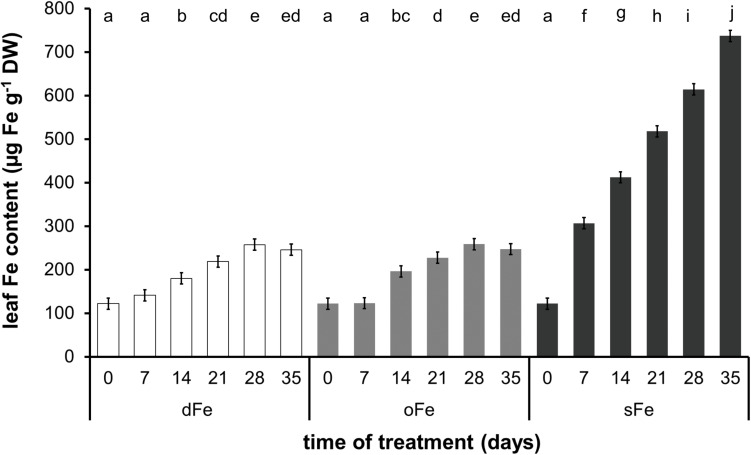
Fe content of the 4th leaves of plants grown on deficient (dFe), optimal (oFe), and supraoptimal (sFe) Fe supply, represented by open, light grey and dark grey columns, respectively, on a dry weight (DW) basis of leaves. Error bars represent SD values. To compare the differences, one-way ANOVA was performed with Tukey–Kramer *post hoc* test on the treatments [*P* < 0.05; *n* = 3 × 3 (biological × technical)].

Regarding the alterations in the Fe content of the 4th leaves (Figure 2), Fe content was stable during the whole time of treatment in dFe and oFe treatments where slight changes did not prove significant. Average values were 194.1.6 ± 55.7 and 195.7 ± 55.3 μg Fe g^–1^ dry weight (DW) for dFe and oFe treatments, respectively. In contrast, sFe treatment induced a significant and tendentious increase in the Fe content of leaves. On the 35th day of treatment, Fe content in sFe leaves was three times higher (737.0 ± 12.8 μg Fe g^–1^ DW) than that in oFe ones.

**FIGURE 3:**
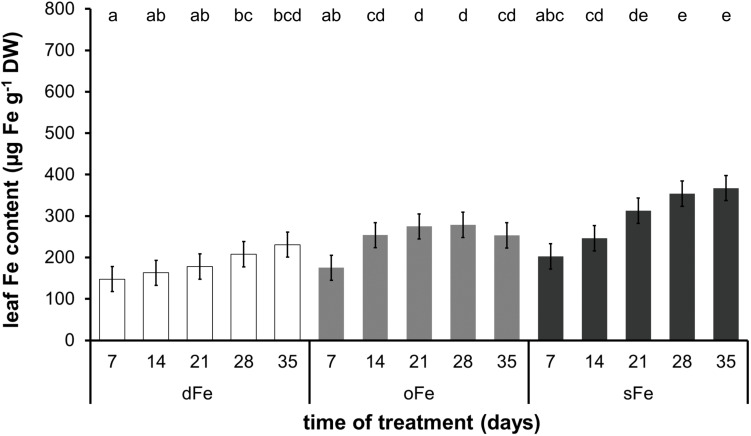
Fe content of the 6th leaves of plants grown on deficient (dFe), optimal (oFe), and supraoptimal (sFe) Fe supply, represented by open, light grey and dark grey columns, respectively, on a dry weight (DW) basis of leaves. Error bars represent SD values. To compare the differences, one-way ANOVA was performed with Tukey–Kramer *post hoc* test on the treatments [*P* < 0.05; *n* = 3 × 3 (biological × technical)].

In contrast to the 4th leaves, Fe content of the 6th ones (Figure 3) showed a tendency of increase during the whole time of treatment in all three groups. Although the Fe content in the 6th leaves of dFe and oFe plants (185.61 ± 33.75 and 247.22 ± 41.94 μg Fe g^–1^ DW, respectively) was comparable to Fe content in the 4th leaves in the corresponding treatments at the end of the treatment time, under sFe treatment, the Fe accumulation in the 6th leaves (296.75 ± 70.81 μg Fe g^–1^ DW) remained significantly lower than that in the 4th leaves of the corresponding plants. Thus, Fe content in the 6th leaves of all three treatments remained comparable.

### Fe Content of Chloroplasts

**FIGURE 4:**
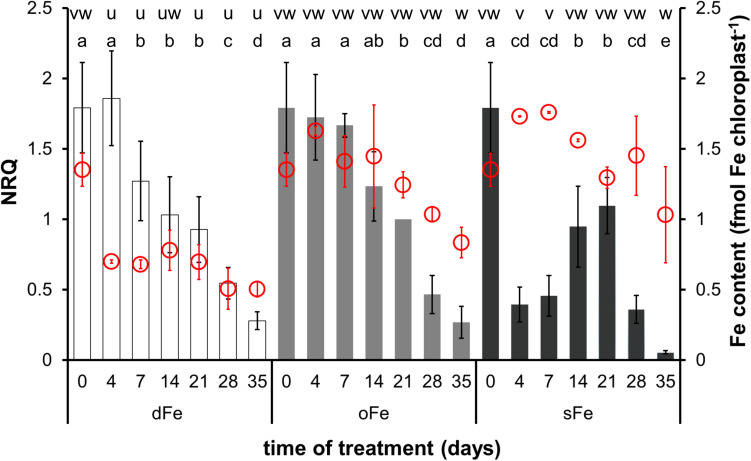
Expression of *BnFRO7* (bar graph, left scale) and Fe content of chloroplasts (scatter plot in red, right scale) in the 4th leaves of plants grown on deficient (dFe), optimal (oFe) and supraoptimal (sFe) Fe supply, represented by open, light grey and dark grey columns, respectively. Error bars represent SD values. To compare differences between times of measurements, one-way ANOVAs were performed with Tukey–Kramer multiple comparison *post hoc* test (expression: *P* < 0.05, *n* = 12; Fe content: *P* < 0.05, *n* = 9), indicated by letters (NRQ: a–e; Fe content: u–w).

In the 4th leaves, chloroplast Fe content decreased in oFe and sFe plants during the time of treatment and in dFe leaves after the 14th day of treatment (Figure 4). Although no difference was found in the Fe content of chloroplasts either at the beginning or at the end of the time of treatment among oFe and sFe treatments, in dFe leaves, the Fe content of chloroplasts was significantly lower during the whole time of treatment.

**FIGURE 5:**
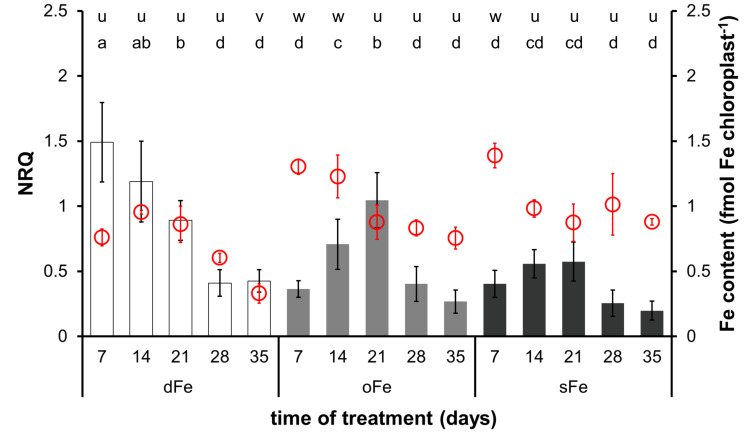
Expression of *BnFRO7* (bar graph, left scale) and Fe content of chloroplasts (scatter plot in red, right scale) in the 6th leaves of plants grown on deficient (dFe), optimal (oFe) and supraoptimal (sFe) Fe supply, represented by open, light grey and dark grey columns, respectively. Error bars represent SD values. To compare differences between times of measurements, one-way ANOVAs were performed with Tukey–Kramer multiple comparison *post hoc* test (expression: *P* < 0.05, *n* = 12; Fe content: *P* < 0.05, *n* = 9), indicated by letters (NRQ: a–d; Fe content: u–w).

In contrast to the 4th leaves, Fe content in the 6th leaves was comparable among treatments during the time of treatment; thus, Fe accumulation in oFe chloroplasts was less retarded than that in the 4th leaves (Figure 5). After reaching the full development of leaves, Fe content in oFe and dFe chloroplasts decreased but in sFe chloroplasts remained stable. Since the decrease was more pronounced in dFe chloroplasts than in oFe on the 35th day of treatment, Fe content in dFe chloroplasts was measured to be significantly lower than that in oFe or even to sFe.

### Identification and Localisation of BnFRO7

**FIGURE 6:**
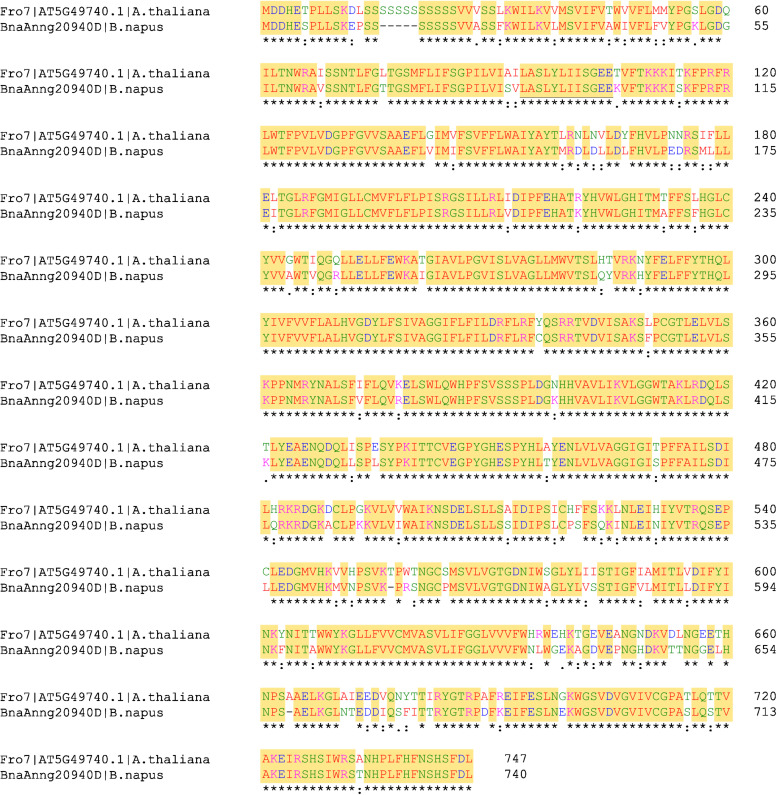
Protein sequence alignment of *Arabidopsis thaliana* FRO7 on the predicted *Brassica napus* FRO7. Yellow highlights indicate identical amino acid positions. Asterisks indicate identical amino acids; colons indicate conservation between groups of strongly similar properties; dots indicate conservation between groups of weakly similar properties. Colour coding of the amino acids: red [small + hydrophobic (including F)], blue (acidic), magenta (alkaline + H) and green (hydroxyl + sulfhydryl + amine + G).

**FIGURE 7:**
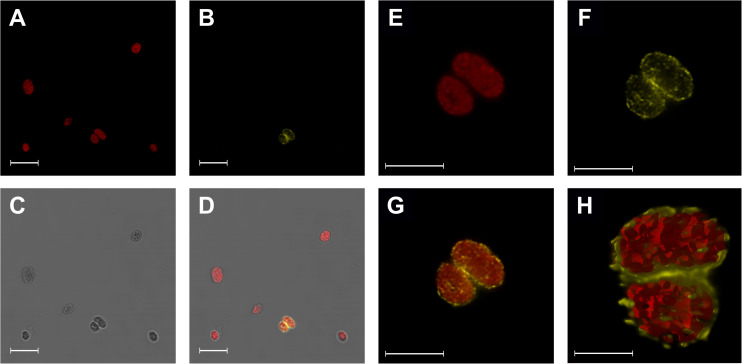
Localisation of BnFRO7-GFP in the *Phaseolus vulgaris* chloroplasts detected by confocal microscopy of immunolabelled chloroplasts decorated with anti-GFP antibody. **(A–D)** Wild type and BnFRO7-GFP containing chloroplasts; chlorophyll autofluorescence. (**E–G)** High resolution images of BnFRO7-GFP containing chloroplasts. **(H)** 3D reconstruction. Reconstruction of the same chloroplasts. Scale bars are 10 μm **(A–D)**, 5 μm **(E–G)**, and 2 μm **(H)**. Colours: chlorophyll (red) and anti-rabbit-IgG Atto 550 conjugated antibody (yellow).

Aligning BnFRO7 to AtFRO7, the sequences showed 85.33% identity and 90.67% similarity (Figure 6). Chloroplast transit peptide was identified in both protein sequences on the N-terminus based on cellular localisation prediction (Supplementary Figure 1). In order to validate the identification of BnFRO7, localisation of GFP fusion construct was studied using a *P. vulgaris* transient expression model (Supplementary Figure 2). In a mixed chloroplast suspension obtained from leaf spots, signal for the immunologically labelled GFP was clearly associated with the chloroplast envelope membranes distinct from Chl autofluorescence, a marker of the thylakoid membranes (Figure 7).

### Expression of *BnFRO7*

In both leaves developed before and during the treatments, the expression of *BnFRO7* was dependent on the Fe nutrition status of the plants. In the 4th leaves, the highest relative transcript abundance was measured on the 4th day of treatment under oFe and dFe treatments (Figure 4). sFe treatment, indeed, induced a rapid effect on the relative transcript amount; thus, on the 4th day of treatment, it was significantly lower than oFe plants. From the 4th day on, the relative transcript amount of *BnFRO7* showed a tendency of decrease in both the 4th leaves of dFe and oFe plants. In contrast, that of sFe plants increased until the 21st day of treatment and turned to be decreasing only thereafter. Nevertheless, peak expression in the 4th leaves of sFe plants remained significantly below that of dFe and oFe plants. Thus, sFe treatment not only decreased the expression of *BnFRO7* in general but also altered the pattern of its relative transcript amount in time.

In comparison to the 4th leaves, the relative transcript amount of *BnFRO7* was sensitive to Fe nutrition in the 6th leaves (Figure 5) where the time-scale pattern in the relative transcript amount in oFe leaves was more similar to sFe plants, including the shift in the peak expression towards the 21st day of treatment. A negative correlation was found between Fe nutrition and the highest relative transcript abundance of *BnFRO7* in the 6th leaves: the higher the Fe nutrition, the lower the relative transcript amount at its peak was found.

### Fe Uptake of Chloroplasts

**FIGURE 8:**
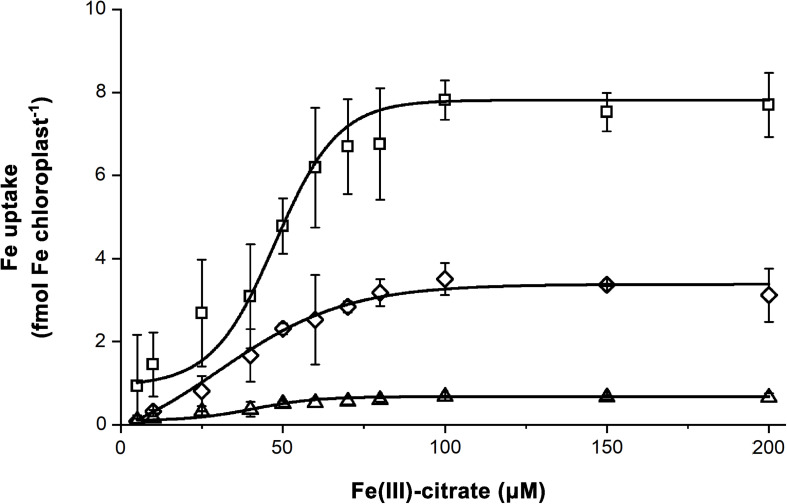
Saturation of the Fe uptake of chloroplasts isolated from leaves of plants grown on Fe deficient (dFe—triangle), optimal (oFe—square) and supraoptimal Fe supply (sFe—diamond). Error bars represent SD values (*n* = 12). Curves represent Boltzmann sigmoidal fits on datasets (*n* = 12).

Iron uptake assays were performed on chloroplasts isolated from leaves developed under dFe, oFe, and sFe treatments, respectively. *In vitro* Fe uptake assays were performed using Fe(III)-citrate as substrate. To characterise the Fe uptake of chloroplasts, Boltzmann fits were applied (Figure 8). Both dFe and sFe treatments decreased the Fe uptake of chloroplasts represented by the decrease in the v_*max*_ of the uptake (Table 2). However, sFe treatment not only caused a decrease in v_*max*_ but also shifted the K_*M*_ of the saturation in comparison to dFe and oFe treatments. This alteration in K_*M*_ indicates a decrease in the substrate affinity. To validate the Fe uptake of chloroplasts, the expression of Fe–S cluster biogenesis machinery SUFB component *BnABCI8* was also studied. Although in the 4th leaves the relative transcript amount of *BnABCI8* showed no significant alterations in the time-scale expression pattern among the Fe nutrition schemes (Supplementary Figure 3), significant and constant repression of this element was measured in leaves developed under Fe restriction (Supplementary Figure 4). In comparison, under oFe and sFe nutrition, the relative transcript amount of *BnABCI8* showed a peak in leaves in parallel to finishing the area growth.

**TABLE 2:** Parameters of the chloroplast Fe uptake activity calculated as Boltzmann sigmoidal fit on Fe acquisition activity of chloroplasts in the function of substrate concentration.

	dFe	oFe	sFe
K_*M*_ [μmol Fe(III)-citrate]	46.37 ± 13.26^*a*^	47.0 ± 2.60^*a*^	30.22 ± 5.43^*b*^
v_*max*_ (fmol Fe chloroplast^–1^)	0.68 ± 0.15^*a*^	7.81 ± 0.16^*b*^	3.38 ± 0.01^*c*^

*To compare the differences, one-way ANOVAs were performed with Tukey–Kramer post hoc test on kinetic parameters (P < 0.001; n = 12).*

### FCR Activity of cIE Vesicles

**FIGURE 9:**
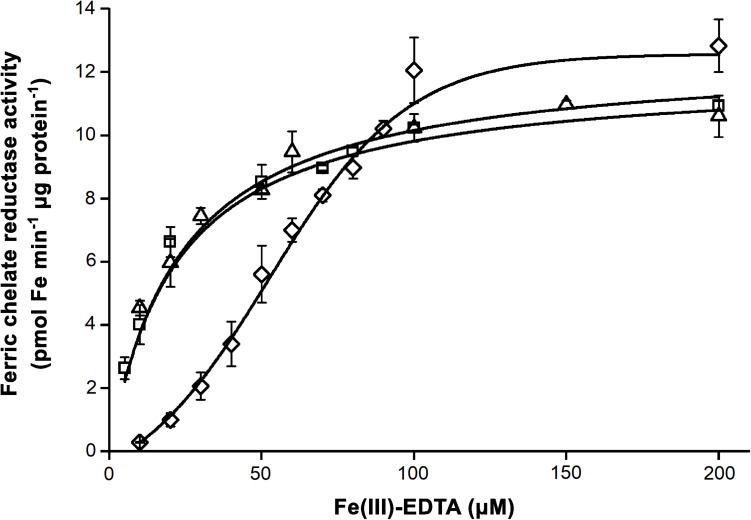
Ferric chelate reductase (FCR) activity of chloroplast inner envelope (cIE) vesicle fractions isolated from leaves of *Brassica napus* grown under Fe deficient (dFe—triangle), optimal (oFe—square) and supraoptimal Fe supply (sFe—diamond). Boltzmann sigmoidal fit represents the saturation of the FCR activity. Error bars represent SD values.

cIE membrane fractions were collected from the 1.0/0.8 M sucrose gradient interface of the isolation processes. As indicated by the strong staining against IEP37 in immunoblot assays, fractions contained cIE vesicles, whereas no apoLHCII, a marker of thylakoid contamination, was detected (Supplementary Figure 5). Purified cIE vesicles were subjected to FCR assays. Fe nutrition of the plants significantly affected the FCR activity (Figure 9). Although the v_*max*_ was not affected in any treatments (Table 3), sFe nutrition induced a significant shift in the substrate affinity compared to dFe and oFe treatments (twofold increase was calculated in the K_*M*_ in sFe samples). Kinetic parameters for FCR reactions in dFe and oFe samples did not differ significantly.

**TABLE 3:** Parameters of the ferric chelate reductase (FCR) activity of the inner envelope fraction of chloroplasts calculated as Boltzmann sigmoidal fit on enzyme activity data in the function of substrate concentration.

	dFe	oFe	sFe
K_*M*_ [μmol Fe(III)-EDTA]	23.46 ± 3.03^*a*^	22.07 ± 2.87^*a*^	52.52 ± 2.36^*b*^
v_*max*_ (pmol Fe min^–1^ μg protein^–1^)	12.54 ± 0.40^*a*^	11.99 ± 0.34^*a*^	12.58 ± 0.53^*a*^

*To compare the differences, one-way ANOVAs were performed with Tukey–Kramer post hoc test on the treatments and affinity components (P < 0.05; n = 12).*

## Discussion

Iron allocation to the chloroplasts is under a strict control. Since Fe is a redox active transition metal, in the presence of ROS, it can participate in Fenton reactions initiating oxidative stress (Shcolnick and Keren, 2006). Previously, mutation of *AtSIA1* and *AtOSA1*, homologues to *Abc1-like* genes, functioning in the biosynthesis of Fe-containing cytochrome *b_6_/f* complexes was shown to lead to a massive Fe incorporation disorder also inducing oxidative stress (Manara et al., 2014). Kim and Jung (1993) showed that Fe excess in chloroplasts leads to photosensitivity and light-induced photoinhibition. Thus, functional chloroplasts cannot serve as stable Fe storage and maintain a strictly controlled Fe status (Solti et al., 2012). Moreover, plants alter the intracellular localisation of Fe as a reaction to altered Fe availability to balance metabolism (Vigani et al., 2013).

### Fe Management of Leaves and Chloroplasts

In a *B. napus* model, Fe content of leaves and chloroplasts was sensitive to the Fe nutrition of the plants both in already developed and in yet developing leaves. Although Fe deficiency caused no alteration in the leaf Fe content, sFe nutrition led to a significant Fe accumulation in leaves. However, this Fe accumulation did not affect the Fe content of chloroplasts that remained unaltered in comparison to plants of optimal Fe nutrition suggesting a vacuolar or apoplastic Fe accumulation. In turn, mung bean (*Vigna radiata*) was previously shown to accumulate a considerable amount of Fe, especially in the thylakoids under a strong excess of Fe (Kim and Jung, 1993). Nevertheless, the rice (*O. sativa*) vacuolar Fe transporter OsVIT1 was shown to be up-regulated under Fe excess together with the decreased allocation of Fe into the mitochondria (Bashir et al., 2011). Thus, strategies for Fe allocation may vary among plant taxa and are highly dependent on the Fe nutrition demand. Indeed, available data are limited on intracellular Fe allocation as a response to the slight surplus of Fe.

Iron homeostasis of chloroplasts is sensitive to the Fe nutrition status of the plant individual. In the reduction-based Fe uptake of chloroplasts, *Arabidopsis* FRO7 is essential. Knocking down its function resulted in a significant decrease of the chloroplast Fe accumulation and lethality under alkaline conditions (Jeong et al., 2008). We identified *B. napus* orthologue of *AtFRO7* based on sequence similarities, the existence of chloroplast targeting peptide and the incorporation of BnFRO7-GFP fusion construct to the chloroplast envelope. In leaves developed before the alterations in the Fe nutrition, the expression of *BnFRO7* was only sensitive to the slight surplus of Fe; thus, exposure to 100 μM Fe nutrition caused a prompt and significant decline in its relative transcript amount. Since during this 1 week of sFe nutrition Fe accumulated only in leaves but not in chloroplasts, the chloroplast Fe uptake machinery proved to be highly sensitive and thus strictly regulated by the available Fe. Solti et al. (2012) indicated that in parallel to the increase in the Fe content in *Beta vulgaris* chloroplasts, Fe uptake decreases to zero at around 4.5 mM internal Fe concentration of chloroplasts. In contrast to leaves developed before the altered Fe nutrition, in leaves developed during the treatments, the Fe content of leaves and chloroplasts was slightly affected. Indeed, the expression of *BnFRO7* was strongly altered. Previous studies reported no significant alterations in the expression of chloroplast FRO upon Fe deficiency, in contrast to root *FRO* genes (Mukherjee et al., 2006; Martín-Barranco et al., 2020). In our measurements, expression of chloroplast *FRO* was not only dependent of Fe nutrition of the plants during leaf development, but an altered pattern in the function of leaf development was found as a response for altered Fe status. Increase in the *BnFRO7* expression was found to be parallel to the development of the photosynthetic apparatus and thus the requirement for Fe in chloroplasts (Nishio and Terry, 1983; Solti et al., 2016; Pham et al., 2020). Moreover, the suppressing effect of surplus Fe nutrition on the expression of *BnFRO7* independently on the developmental status of the leaves is evident. Thus, chloroplast-born and cytoplasmic Fe signals together effectively suppress chloroplast Fe uptake by down-regulating *FRO7*. Indeed, only pieces of the signals of Fe status in mesophyll cells and particularly in chloroplasts are known (Vigani et al., 2013). *Vaccinium corymbosum* LON1 protease was reported to play a role in maintaining the Fe homeostasis in plastids increasing plastidial Fe content under insufficient Fe nutrition (Zhong et al., 2019). ROS are important retrograde signalling compounds that are suggested to be involved in monitoring the chloroplast Fe status and involved in Fe status monitoring processes (Vigani et al., 2013; Balparda et al., 2020; Unal et al., 2020). Nitric oxide (NO) has also been reported to regulate Fe homeostasis in chloroplasts (Arnaud et al., 2006; Touraine et al., 2012). *S*-nitrosoglutathione (GSNO) formed in the reaction of NO with reduced glutathione may serve as a NO donor (Leterrier et al., 2011). Diglutathionyl-dinitrosyl-Fe complexes were also reported to have NO signalling functions (Le et al., 2019). GSNO reductase (GSNOR) contributes to the decrease of GSNO level. Long-term Fe deficiency led to a significant increase in the *S*-nitrosothiol (SNO) content of cells (Wen et al., 2019). Under oFe nutrition, overexpression of GSNOR decreased the expression of *Ferritin2*, *PIC1*, *FRO7* and *VIT* of these genes in mature leaves of *Arabidopsis* plants (Wen et al., 2019). Thus, GSNO is a potential candidate for the retrograde signalling of the Fe status of chloroplasts. Nevertheless, the origin for the signal suppressing chloroplast Fe acquisition has not been clarified yet. Regarding the Fe signalling of the eukaryotic part of the plant cell, Brutus (BTS) and BTS-Like ubiquitin ligases mediate Fe deficiency responses (Hindt et al., 2017). Based on the similarities to the mammalian F-box/LRR protein 5, their stability is suggested to be regulated by the binding of Fe (Rodríguez-Celma et al., 2019). Expression of *BTS* increases in shoot tissues under Fe deficiency (Rodríguez-Celma et al., 2013; Hindt et al., 2017). Targets of BTS were identified as bHLH104, bHLH115, and bHLH105 (ILR3) (Selote et al., 2015). Nevertheless, BTS is also suggested to be involved in other signalling processes based on the sensitivity to ROS (Tripathi et al., 2018). BTS was shown to be involved in the regulation of *FRO3* since it remained constitutively activated in *bts-3* mutant line (Hindt et al., 2017). Nevertheless, discoveries on the regulation network mainly focus on Fe deficiency, and thus hardly any information can be found on the signalling of the excess of Fe.

### Alterations in the Fe Allocation

Iron allocation at leaf and chloroplast levels was different, predominantly in developed leaves. While Fe content in leaves remained stable under dFe and oFe nutrition leaves after reaching full maturity, Fe content of the chloroplasts, together with the expression of *BnFRO7*, declined. The decrease in the Fe content per chloroplasts (together with a previously reported decline in the photosynthetic activity, Pham et al., 2020) indicates that Fe release mechanisms should exist that contribute to the export of Fe from plastids. Recently, chloroplast NEET protein was found to be involved in the export of Fe–S clusters to cytoplasmic Fe–S proteins. Disrupting NEET function resulted in enhanced ROS generation and Fe over-accumulation (Zandalinas et al., 2019). RNAi of At-NEET resulted in elevated sensitivity to low and decreased sensitivity to high Fe nutrition (Nechushtai et al., 2012; Lu, 2018). The tendentious decrease in the relative transcript amount of *BnFRO7* in the 4th leaves of dFe and oFe plants indicates that FRO7 primarily operates during the development of leaves when the incorporation of Fe into Fe–S clusters and heme together with the biogenesis of the photosynthetic apparatus requires the majority of Fe supply (Morrissey and Guerinot, 2009). Decrease in the *BnFRO7* expression is in line with the recently indicated decrease in the transcription of *SUFB*, a key component of the chloroplast Fe–S cluster assembly complex (Kroh and Pilon, 2020a). Our measurements also underlined that initiation of senescence also suppressed the expression of *SUFB* component *BnABCI8* indicating that the reduction-based chloroplast Fe acquisition is strongly coupled to the incorporation of Fe particularly into Fe–S clusters (Solti et al., 2012). In the 6th leaves of dFe plants, suppression of *BnABCI8* indicated that the reciprocal connection between decreased Fe allocation and uptake to the chloroplasts and lowered chloroplast Fe accumulation also impacts the Fe–S cluster assembly.

### Regulation of Chloroplast Fe Acquisition

The saturation of the requirement for Fe, especially during the leaf development, led to a suppression of the reduction-based Fe uptake of chloroplasts. This suppression is also supported by the significant increase in the K_*M*_ of FCR reaction in sFe cIE membranes compared to that of oFe and dFe samples, indicating a reduction in the affinity for substrates. This loss in the substrate affinity contributed to the decreased Fe uptake of isolated chloroplasts (decrease in the v_*max*_). This indicates a protein level and supposedly chloroplast-born regulation of FRO7. Although there are no direct literature data on the protein level regulation of chloroplast FRO to date, NO production that has been previously confirmed in chloroplasts under Fe excess (Touraine et al., 2012; Kolbert et al., 2019) may be involved in this allosteric regulation. *S*-nitrosylation of Cys residues of enzymes, such as the plasma membrane NADPH oxidase, has such a regulative role (Yun et al., 2011; Begara-Morales et al., 2014). Thus, further investments are required to clarify the role of protein modifications in chloroplast Fe homeostasis. *Arabidopsis* root FRO2 taking part in a tripartite protein interaction with the H^+^-ATPase AHA2 and the metal transporter IRT1 was shown not to be regulated by protein modification since its ubiquitination proved to be independent of the presence of non-iron divalent metals, but the phosphorylation of IRT1 was a driving force of the dissociation of the complex (Martín-Barranco et al., 2020). Although protein level interactions of FRO7 have not been studied so far, the lack of accumulation of Fe^2+^ at light-induced acquisition suggests a close collaboration of FRO7 to uptake transporters (Solti et al., 2012; Vigani et al., 2019). Although dFe nutrition did not alter the FCR activity (no increase was found in the affinity) and *BnFRO7* expression in general, Fe uptake of dFe chloroplasts significantly decreased compared to that of oFe. Since FRO7 requires NADPH for the FCR activity that originates from the operation of the photosynthetic electron transport chain (Solti et al., 2014), the decreased Fe uptake is connected to the decreased performance. Taken together, suboptimal Fe limits the available Fe for the chloroplast uptake by the disturbed biosynthesis of the photosynthetic apparatus (Basa et al., 2014). This also suggests that reduction-based Fe uptake of chloroplasts has a primary dominance that cannot be bypassed by any other (if there is any) transport mechanisms effectively.

## Conclusion

In conclusion, both Fe deficiency and sFe nutrition led to a holistic suppression of the reduction-based Fe uptake of chloroplasts; thus, the regulation of the operating component FRO7 has a fundamentally different pattern in comparison to the well characterised root FROs. In contrast to root tissues where the lack of Fe induces a massive expression and activity of the FRO, BnFRO7 is less sensitive to the deprivation of Fe. Saturation of the Fe requirement led, indeed, to a suppression of *BnFRO7* expression and reduction in the substrate affinity of FRO7 in the chloroplast inner envelope membrane indicating that both transcript and activity level regulation mechanisms are involved in the suppression under supraoptimal Fe nutrition that protects chloroplasts against Fe overloading.

## Data Availability Statement

The original contributions presented in the study are included in the article/Supplementary Material, further inquiries can be directed to the corresponding author/s.

## Author Contributions

ÁS designed and supervised the study. BT and BK performed determination of leaf Fe content. MS-K performed chloroplast iron determination. BM and ÁS performed iron uptake assays of the isolated chloroplasts. MS-K, BC, BG, FF, and BM performed chloroplast envelope membrane isolations and *in vitro* ferric chelate reductase assays. BB, FB, and KS identified BnFRO7 sequence, and designed primers for expression analysis. HZ made BnFRO7-GFP fusion construct and transient expressing plant model. AK, TV, and EB performed confocal fluorescence microscopy studies. MS-K, KS, and SF performed expression analysis studies. MS-K, BM, HZ, SF, and ÁS wrote the manuscript. All authors critically reviewed the manuscript.

## Conflict of Interest

BB and FB were employed by company RT-Europe Non-profit Research Ltd. KS was employed by company Carlsbad Research Organization Center Ltd. The remaining authors declare that the research was conducted in the absence of any commercial or financial relationships that could be construed as a potential conflict of interest.

## Footnotes

1 http://brassicadb.org/brad

2 https://www.arabidopsis.org

3 https://www.ebi.ac.uk/Tools/msa/clustalo/

4 https://www.bioinformatics.org/sms2/ident_sim.html

5 http://www.cbs.dtu.dk/services/ChloroP/

## Supplementary Material

Supplementary Figure 1 Transient expression of BnFRO7-GFP construct in first leaves of common bean.

Supplementary Figure 2 Presence of chloroplast transit peptide in BnFRO7.

Supplementary Figure 3 Relative transcript amount of *BnABCI8* in 4th leaves.

Supplementary Figure 4 Relative transcript amount of *BnABCI8* in 6th leaves.

Supplementary Figure 5 Purity of chloroplast inner envelope isolates.

Supplementary Material 1 Element content data of leaves.

## Abbreviations

dFe: iron deficiency
FCR: ferric chelate reductase
oFe: optimal iron nutrition
sFe: supraoptimal iron nutrition.
