# Supplementary material for: Supraoptimal Iron Nutrition of Brassica napus Plants Suppresses the Iron Uptake of Chloroplasts by Down-Regulating Chloroplast Ferric Chelate Reductase
Source: Front Plant Sci. 2021 May 20;12:658987. doi: 10.3389/fpls.2021.658987 (PMC8172622; doi:10.3389/fpls.2021.658987)
Supplement: Supplementary Figure 1 — Transient expression of BnFRO7-GFP construct in first leaves of common bean. [file Data_Sheet_1.PDF]

CENTERFO RBIOLOGI CALSEQU ENCEANA LYSIS CBS

ChloroP 1.1 Server - prediction results

Technical University of Denmark

```
### chlorop v1.1 prediction results ##########################
Number of query sequences: 3

Name                  Length       Score  cTP        CS-    cTP-
                                                    score  length
-----------------------------------------------------------------
Fro2_AT1G01580.1_A.t     725        0.451   -     -1.232      80
Fro7_AT5G49740.1_A.t     747        0.557   Y      3.050      28
Fro7_BnaAnng20940D_B     740        0.508   Y      3.234      22
-----------------------------------------------------------------
```

Explain the output. Go back.

Supplementary Figure 1. Prediction for the presence of chloroplast transit peptides (cTP) in *AtFRO2* (*At1g01580.1_A.t*; accessed in NCBI; negative control, certified root plasma membrane localisation), *AtFRO7* (*At5g49740.1_A.t*; accessed in NCBI; positive control of certified chloroplast envelope membrane localisation) and predicted *BnFRO7* (*BnaAnng20940D_B*; accessed in NCBI) by ChloroP 1.1 Server (Technical Univeristy of Danemark). Results indicate whether target sequences are predicted to contain cTP; Y indicated positivity of cTP prediction; "–" indicates negative results of prediction. The prediction of the presence of cTP is solely based on second step score value. CS-score indicates results of MEME scoring matrix for the suggested cleavage site. The cTP-length value indicates the predicted length of the presequence in number amino acids.

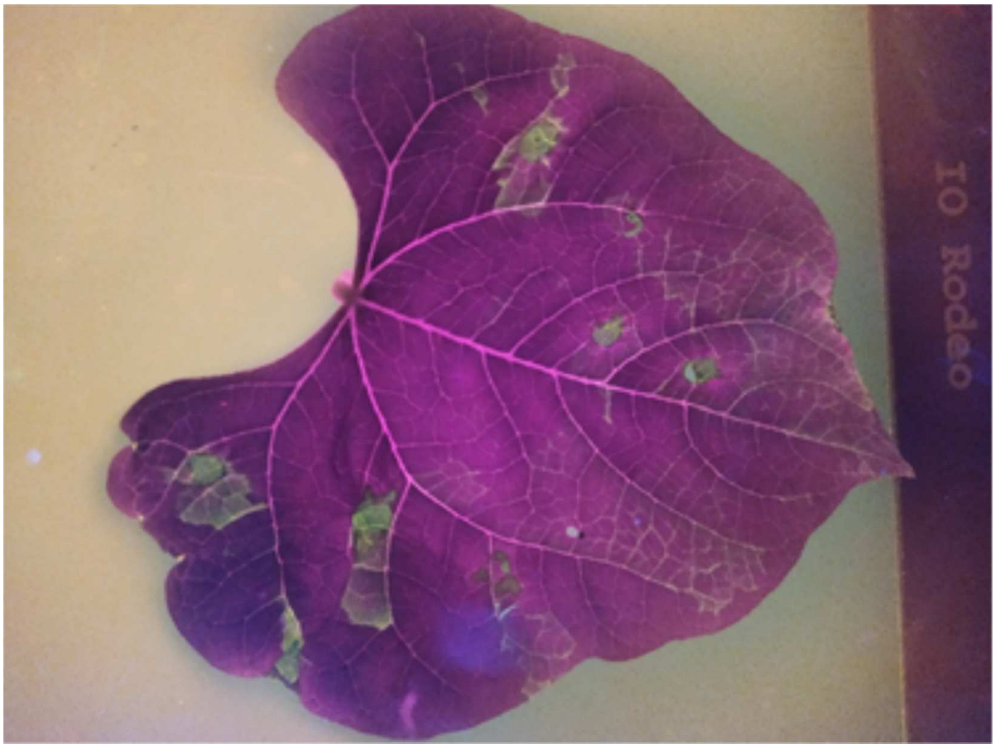

Supplementary Figure 2. Transient expression of BnFRO7-GFP construct in first leaves of common bean (*Phaseolus vulgaris* L. var. *nanus* cv. Borlotto) after 5 days of spot infiltration. Excitation was carried out by an E=474[±12] nm blue light source in IORodeo Midi blue LED transilluminator. Green fluorescence of GFP was detected as F_{max}=510 nm green fluorescent dots appeared macroscopically. Image was taken using 64MP AI Quad Camera (Xiaomi Inc., China).

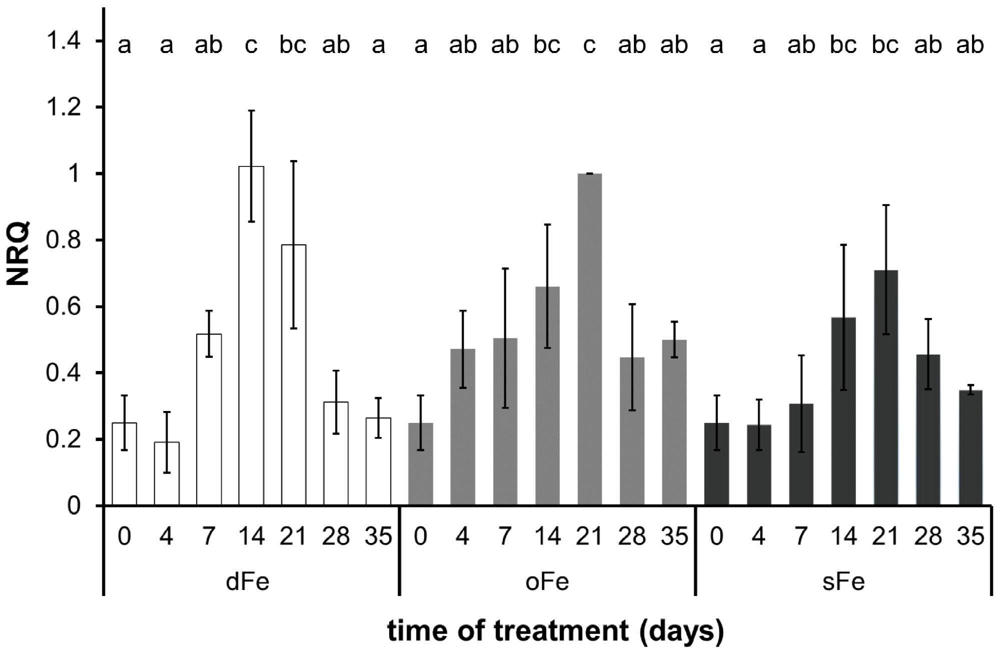

Supplementary Figure 3. Relative transcript amount of *BnABCI8* in 4th leaves. Error bars represent SD values. To compare differences between times of measurements, one-way ANOVAs were performed with Tukey-Kramer multiple comparison *post hoc* test; $P<0.05$, $n=12$. Groups are indicated by lowercase letters.

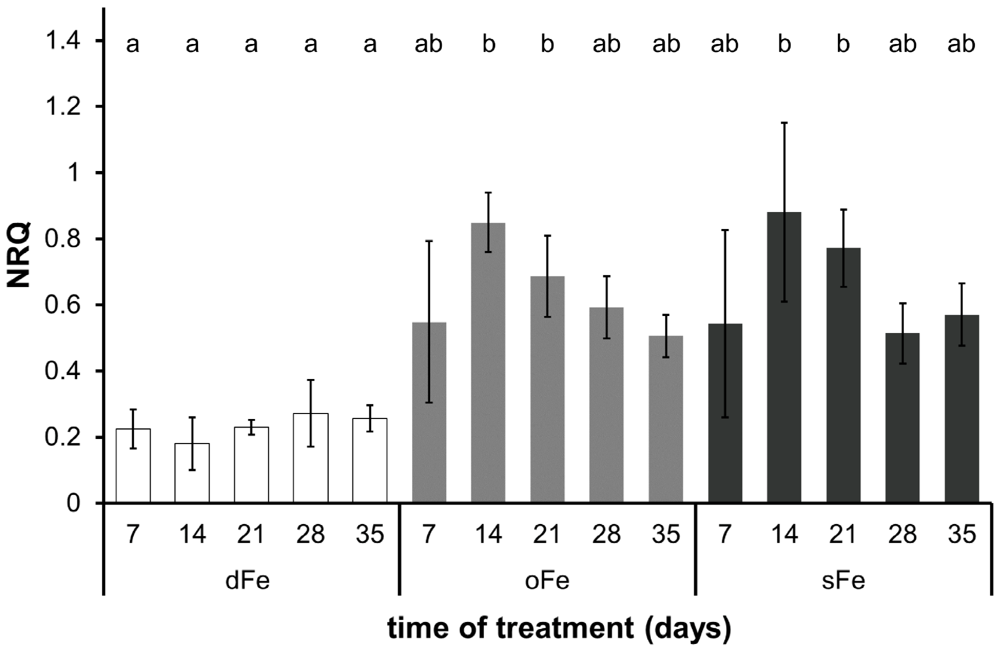

Supplementary Figure 4. Relative transcript amount of *BnABCI8* in 6th leaves. Error bars represent SD values. To compare differences between times of measurements, one-way ANOVAs were performed with Tukey-Kramer multiple comparison *post hoc* test; $P<0.05$, $n=12$. Groups are indicated by lowercase letters.

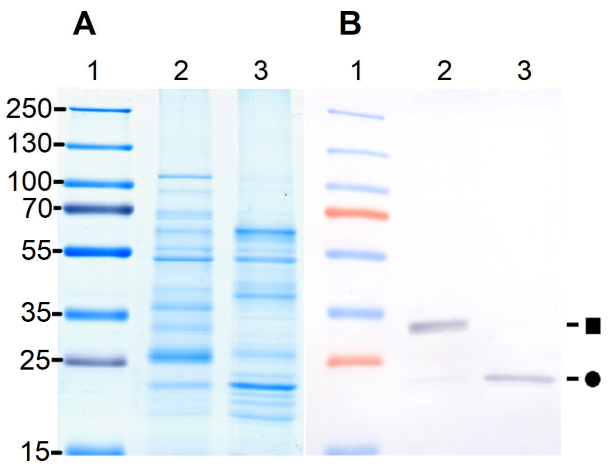

Supplementary Figure 5. (A) Coomassie-stained solubilized proteins on polyacrylamide gel and (B) combined immunoblot against chloroplast inner envelope protein 37 (IEP37) and apoLhcII (B). Lanes are: 1 – Fermentas Page Ruler Pre-stained Protein SM0671 (Thermo-Fisher Scientific) pre-stained molecular weight standard, 2 - chloroplast inner envelope (cIE) fraction, 3 – thylakoid fraction. Marks are: square – IEP37; circle – apoLhcII. Lanes on protein gels and immunoblots were loaded with 15 μg solubilized protein.
